# A Population-Based Model of the Temporal Memory in the Hippocampus

**DOI:** 10.3389/fnins.2018.00521

**Published:** 2018-08-07

**Authors:** Sorinel A. Oprisan, Mona Buhusi, Catalin V. Buhusi

**Affiliations:** ^1^Department of Physics and Astronomy, College of Charleston, Charleston, SC, United States; ^2^Interdisciplinary Program in Neuroscience, Department of Psychology, Utah State University, Logan, UT, United States

**Keywords:** hippocampus, topological map, scale invariance, time cells, computer simulations, neural networks

## Abstract

Spatial and temporal dimensions are fundamental for orientation, adaptation, and survival of organisms. Hippocampus has been identified as the main neuroanatomical structure involved both in space and time perception and their internal representation. Dorsal hippocampus lesions showed a leftward shift (toward shorter durations) in peak-interval procedures, whereas ventral lesions shifted the peak time toward longer durations. We previously explained hippocampus lesion experimental findings by assuming a topological map model of the hippocampus with shorter durations memorized ventrally and longer durations more dorsal. Here we suggested a possible connection between the abstract topological maps model of the hippocampus that stored reinforcement times in a spatially ordered memory register and the “time cells” of the hippocampus. In this new model, the time cells provide a uniformly distributed time basis that covers the entire to-be-learned temporal duration. We hypothesized that the topological map of the hippocampus stores the weights that reflect the contribution of each time cell to the average temporal field that determines the behavioral response. The temporal distance between the to-be-learned criterion time and the time of the peak activity of each time cell provides the error signal that determines the corresponding weight correction. Long-term potentiation/depression could enhance/weaken the weights associated to the time cells that peak closer/farther to the criterion time. A coincidence detector mechanism, possibly under the control of the dopaminergic system, could be involved in our suggested error minimization and learning algorithm.

## 1. Introduction

Spatial and temporal dimensions are fundamental for orientation, adaptation, and survival of organisms. Hippocampus has been identified as the main neuroanatomical structure involved both in space and time perception and their internal representation. It has been hypothesized that hippocampus may be in fact involved in conceptual understanding of many other dimensions (Schapiro et al., 2015; Schiller et al., 2015). While hippocampus encodes and processes information regarding spatial location and temporal durations, the same or similar computational structures could be used for encoding and processing the “state” of the brain associated to more abstract tasks than spatial location, such as extracting patterns from apparently random events (Garvert et al., 2017). It has been suggested that the same or a similar computational algorithm that establishes a metric space for the abstract dimension of time could be employed to create metric spaces and ordered categories for cognitive maps that relate other abstract concepts (Howard et al., 2014).

**Temporal Dimension and Time Cells**. One of the most used experimental paradigms when investigating temporal perception is the treadmill running, which allows precise correlation of neural firing with the spatial location and temporal duration. *In vivo* recordings from hippocampus and entorhinal cortex showed that neurons ramp-up their firing only at specific temporal locations during a behavioral tasks (Pastalkova et al., 2008; MacDonald et al., 2011; Kraus et al., 2013; Wang et al., 2015). This activity is similar to the hippocampus “place cells” that ramp-up their firing only when the subject is in a specific spatial location (O'Keefe, 1976; O'Keefe and Recce, 1993; Mathis et al., 2012). This is the reason the hippocampus cells that fire at a specific time during a behavioral test are called “time cells.” Single-cell recordings from hippocampus suggested a clear correlation of the firing rate of the time cells with the to-be-timed duration (MacDonald et al., 2011). They found that different time cells selectively and repeatably peak at specific moments during the to-be-timed duration (MacDonald et al., 2011). In similar experiments involving a reward after a specific delay time, it has been found also that neurons in the rodent hippocampus selectively fired at specific times after the beginning of a delay period (Pastalkova et al., 2008). It has been shown that some time cells are involved in timing absolute durations whereas others fire relative to a specific temporal marker (MacDonald et al., 2011). Neurons that fired during the to-be-timed interval were typically striking in their selectivity to specific moments in the time interval. These time cells fire at successive moments within a temporally defined period. A hallmark of the time cells experiments is that the spread of the firing interval, i.e., the width of the Gaussian-like activity, for each time cell is proportional to the time of the peak activity. Additional experimental measurements also confirmed the proportional spread in time fields of time cells for longer durations (Kraus et al., 2013; Howard et al., 2014). Such a response reflects a cellular-level accumulated error in timing from the outset of the to-be-timed interval similar to the scalar property in behavioral experiments of interval timing (Gibbon and Church, 1984; Gibbon et al., 1984). This paper suggests a possible bridge between the cellular-level, experimentally measured, proportionality relationship between the spread of the time field and the time of peak activity of the time cell (Pastalkova et al., 2008; MacDonald et al., 2011; Kraus et al., 2013; Howard et al., 2014) and the scalar property of interval timing. The linear relationship between the peak time and the spread of the Gauss-like, i.e., the error of time estimation increases linearly with the to-be-timed interval, which has been measured in behavioral experiments, such as the peak interval procedure (Buhusi and Meck, 2005, 2006; Buhusi et al., 2006, 2009; Buhusi and Oprisan, 2013).

**Similarities and Differences Between This Novel Population-Based Topological Map of the Hippocampus and Other Timing Models**. This new model of temporal memory storage in the hippocampus is based on two core hypotheses: (1) the existence of a spatially ordered memory of temporal durations (topological map) in the hippocampus, and (2) the time cells form a temporal basis for time perception. This model of the long-term memory block could also be included in more comprehensive timing models, such as the Scalar Expectancy Theory (SET) (Gibbon, 1977; Church, 1984; Church and Broadbent, 1990, 1991; Church et al., 1994, 1998; Gibbon et al., 1997), Striatal Beat Frequency (SBF) (Buhusi and Meck, 2005, 2009, 2010; Oprisan and Buhusi, 2011, 2013, 2014; Buhusi and Oprisan, 2013; Buhusi et al., 2016; Oprisan et al., 2018), the Behavioral theory of Timing (BeT) (Killeen and Fetterman, 1988, 1993; Bizo and White, 1994), or the Learning-to-Time (LeT) model (Machado, 1997; Machado and Silva, 2007; Machado et al., 2007). Without going into an extensive literature review of the SET model (see Gallistel, 1990; Gibbon, 1991; Church, 2003 and references therein), we only briefly mention here the key functional building blocks of the SET model that other theoretical models of time perception preserved and expanded upon. The SET model postulates the existence of an internal clock composed of three blocks: a pacemaker-accumulator, a memory, and a comparator. The role of the pacemaker is to generate clock pulses that are added up by the accumulator block. The accumulator is reset at the beginning of every trial, presumably through a dopamine-mediated mechanism. The long-term memory block stores the value of the accumulator at the reinforcement time in each trial. After a large number of trials, the long-term memory contains a Gaussian distribution of reinforcement times approximating the criterion time. According to the SET model, the decision whether or not to respond in a test trial is determined by the ratio between a randomly drawn value of the criterion time from its multiple, Gauss distributed, copies stored in the long-term memory and the current value of the accumulator. When the ratio computed by the comparator block crosses a given threshold, the animal changes its response from a low to a high rate. Our previous topological map model of the hippocampus also stored a set of reinforcement times in the long-term memory (Oprisan et al., 2018). Compared to SET, the novelty of the topological map was the hypothesis of a spatially ordered structure of the hippocampus, which allowed us to explain the experimental results on hippocampus lesions.

Both BeT and LeT models require three elements: a series of states, a series of operant responses, and a set of associative links connecting the states to the operant responses. It is convenient to conceptualize the transitions between states as being driven by a pacemaker block (“even though the mathematical models do not strictly require a biological pacemaker that emits pulses” Killeen and Fetterman, 1993). In the BeT and LeT models, a certain number of states may be required to produce an operant response. Although it is “unclear how states relate to measurable behavior or what their neural basis is” (Machado et al., 2007), it is assumed that they are related to behavioral responses through associative weights that have a natural rate of decay and a reinforcement-dependent rate of increase. There is a similarity between our proposed population-based topological map model of the hippocampus and the LeT model: both use a distributed set of weights. In the LeT model, the weights of the associative links connect states to the operant response, whereas in our computational model the weights represent contributions of time cells to the average time field. In the model we present here, we preserved the topological map from Oprisan et al. (2018) and replaced the actual reinforcement times by the weights of the corresponding time cells. This improvement allowed us to define a learning rule for the weights in order to minimize the error signal, which is proportional to the temporal distance between the time of the peak activity for a given time cell and the to-be-learned criterion time.

## 2. Temporal maps in the hippocampus generated by a population of “time cells”

### 2.1. A population-based model for the topological map of the hippocampus

While we successfully modeled the hippocampus lesions with the previously proposed topological maps (Oprisan et al., 2018), the model was not well-connected with neurobiology. Like SET model, it assumed that the hippocampus simply records the reinforcement times with the added twist of a spatially ordered temporal map with increasingly longer durations stored toward the dorsal side of he hippocampus. Additionally, the topological map model assumed that the distribution of memorized times is Gaussian, with more memory cells holding reinforcement times closer to the criterion time. This is a heuristic assumption consistent with the SET model (see Church, 1984; Gibbon and Church, 1984; Gibbon et al., 1984, 1988; Brunner et al., 1997) hypothesis that at the reinforcement time each trials stores in the hippocampus a slightly different value of the to-be-memorized criterion time. In this study, we suggested a novel approach to the existing topological map model. In order to preserve the ability of the new model to match experimental data on hippocampus lesions, we still assumed that a spatially ordered (topological) map exists in the hippocampus. However, in this new model a memory cells no longer stores a single value, i.e., the reinforcement time *t*_*n*_, but rather stores a pointer to an entire object, i.e., a time cell whose activity peaks at the specific time *t*_*n*_. Therefore, here we suggest a new model of the long-term memory based on a possible connection between the hippocampus topological maps and a population of time cells.

**Model Constraint: Scale Invariance of Time Perception**. The experiments on time perception seem to suggest that the time perception error increases linearly with the to-be-timed duration, which is referred as the scalar property (Buhusi et al., 2017; Daniels and Sanabria, 2017). Scalar property has been found in episodic memory (Glenberg et al., 1980; Howard et al., 2008), peak interval timing (Lewis and Miall, 2009), and conditioning (Balsam and Gallistel, 2009). At the same time, scalar property is shared across different species from mice (Malapani and Fairhurst, 2002; Buhusi et al., 2009) to humans (Rakitin et al., 1998). As a result, every time perception model must include an explanation for the origin of the scale invariance.

**Figure 1:**
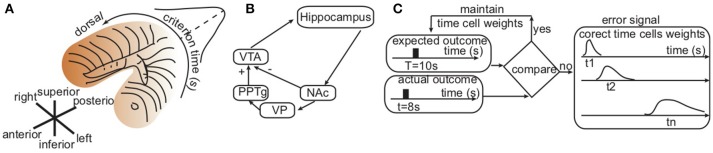
Hippocampal topological map and basal ganglia–hippocampal loops. **(A)** Consistent with hippocampus lesions experiments, the sketch of the hippocampus with color shading suggesting that shorter durations are stored in the ventral area and longer durations orderly stored in the dorsal area. **(B)** Simplified basal ganglia–hippocampal loops with direct projection to the nucleus accumbens (NAc), which performs a decision task by comparing the predicted and the actual outcomes of behavior. If the outcome is as expected, the result is an inhibition (“−”) of ventral tegmental area (VTA). Otherwise, an error correction loop that includes the ventral pallidum (VP) and the pedunculopontine nucleus (PPTg) excites (“+”) VTA. **(C)** Symbolic representation of computations performed by the basal ganglia-hippocampus loops in order to correct the activity of time cells such that the average time field peaks around the to-be-memorized criterion time *T*. An error signal proportional to the distance between the expected peak time *T* and the actual peak times *t*_1_, *t*_2_, …, *t*_*n*_ of individual time cells changes the weights of the contribution of the individual time cells.

**Model Constraint: Peak Time Shift After Hippocampus Lesions**. Hippocampus lesion experiments showed that dorsal hippocampal (DH) lesions induced leftward shifts in peak times (Tam et al., 2015), i.e., toward shorter durations, whereas ventral hippocampal (VH) lesions produced opposed peak shift (Yin and Meck, 2014). Based on the above-cited and similar experimental results, we recently suggested a topological map model of the hippocampus (Oprisan et al., 2018). Briefly, the reinforcement times centered around the desired criterion time learned during successive conditioning trials were modeled as Gaussian random variables. The Gaussian distribution hypothesis was based on existing experimental observations and the justification from the influential SET model (see Church, 1984; Gibbon and Church, 1984; Gibbon et al., 1984, 1988; Brunner et al., 1997). SET assumes that the to-be-learned duration is the result of many training trials that add up to a Gaussian-like response (see Figure 1A). Since lesion experiments suggested a very specific, ordered, arrangement of the memorized durations in the hippocampus (see Tam and Bonardi, 2012a,b; Tam et al., 2013, 2015; Yin and Meck, 2014), we modeled the hippocampus as a spatially ordered map that stores a Gaussian-like distribution of reinforcement times. That topological map model of time perception assumed that the hippocampus serves as a memory storage for elapsed durations with the shorter durations orderly stored toward the ventral side and progressively longer durations stored toward the dorsal side (Oprisan et al., 2018). Such a spatial localization (topological map) of reinforcement times allowed us to both (1) explain experimentally observed effects of hippocampus lesions on interval timing and (2) predict mathematically the relationship between the width and the peak location of the Gaussian-like output function. Without repeating the derivations from Oprisan et al. (2018), we previously showed that the relationship between the width and the peak duration of the output function after the lesions matches the pre-lesion relationship (Oprisan et al., 2018). In other words, our topological map model of the hippocampus preserved the properties of the distribution of memorized durations and only shifted the peak responses proportional to the lesion size and its location relative to the median line of the hippocampus (Oprisan et al., 2018).

**Hypothesis 1: Temporal Boundary**. First, we need to explain how the time cells of the hippocampus know to cover the entire to-be-timed duration. For this purpose, we hypothesize that the mechanism is similar to the one used by the “place cells” of the hippocampus, i.e., place cell firing depends on the position of environmental boundaries (O'Keefe and Burgess, 1996). Similarly, during peak-interval procedure, the mouse learned the “boundaries” of the temporal duration through a series of reinforcement trials. In our current implementation, the temporal boundary of the time cells is three times the criterion time with time cells uniformly distributed over the entire duration.

**Hypothesis 2: Coincidence Detection Dynamically Adjusts Time Cell Firing Rates**. The second assumption of our newly proposed mechanism for generating a Gaussian-like and scale invariant average time field is that dopamine modulation could enhance the activity of certain time cells during reinforcement trials and depressed the activity of others. In order to know which weights to strengthen/weaken, we rely on the existence of a coincidence detector mechanism, such as the one attributed to striatum (Houk, 1995; Houk et al., 1995; Parent and Hazrati, 1995a,b; Harrington et al., 1998; Atallah et al., 2004; Jin et al., 2009; Buhusi et al., 2016), that selectively enhances the activity of those time cells that fire close to the to-be-timed duration. Additional evidence of a possible involvement of the striatum as a coincidence detector is that the cortico-striatal system is involved in “habit” or “procedural” learning. The dopaminergic projections to striatum also support the hypothesis of a modulatory feedback effect that enhances desirable behaviors and suppresses others (Schultz, 2002). It has been suggested that long-term potentiation/depression could reliably modulate the synaptic weights in cortico-striatal loops (Teki et al., 2012). Such dopamine-mediated reinforcement signals could fine-tune the synaptic weights based on repeated trails until the correct criterion time is learned (Gu et al., 2011; Jones and Jahanshahi, 2011). The hippocampus stores representations of individual experiences and seems to carry out a completely different type of memory function than the striatum (Packard et al., 1994; Canal et al., 2005), although some view the two systems as complementary (Atallah et al., 2004). Figure 1B shows a sketch of a possible neurobiological network involving time cells in the hippocampus, the striatum coincidence detector and the dopaminergic reinforcement.

**Possible Basal Ganglia-Hippocampus Loops**. Our suggested simplified network (Figure 1B) uses well-known direct (Voorn et al., 2004) and indirect (Christakou et al., 2004) anatomical connections between the hippocampus and the striatum (see Thierry et al., 2000 for an extensive review of existing pathways). For example, there are known GABAergic projections from the basal ganglia nuclei, the substantia nigra pars reticulata, and the internal part of the globus pallidus to pontine nucei (Parent and Hazrati, 1995a,b). In turn, cholinergic projections from the pedunculopontine nucleus directly (Datta et al., 1998; Silkis, 2008) and indirectly (through thalamic nucleus reuniens Vertes, 2001; McKenna and Vertes, 2004) control the activity of the hippocampus. Among many other identified neural loops, neurons of the CA1 hippocampus area project mainly to the nucleus accumbens (NAc) of the striatum. This nucleus projects into the thalamic nuclei of the middle line through the ventral pallidum, and these thalamic nuclei project back into the NAc and hippocampus (Groenewegen et al., 1999).

A simplified loop (Penner and Mizumori, 2012) that involves a direct projection from the hippocampus to the nucleus accumbens (NAc) of the ventral striatum seems to perform a coincidence detection task by comparing the predicted and the actual outcomes of behavior and either (1) inhibits (marked by “−” in Figure 1B) ventral tegmental area (VTA) if the action matches the predicted behavior, or (2) excites (“+”) VTA in the case of a mismatch. One possible realization of such an indirect path is via ventral pallidum (VP) and the pedunculopontine nucleus (PPTg) (Penner and Mizumori, 2012). One possible effect of this excitatory input to VTA is to bias it more closely to a bifurcation point where is more sensitive to subsequent reward information (see Figure 1B).

**Figure 2:**
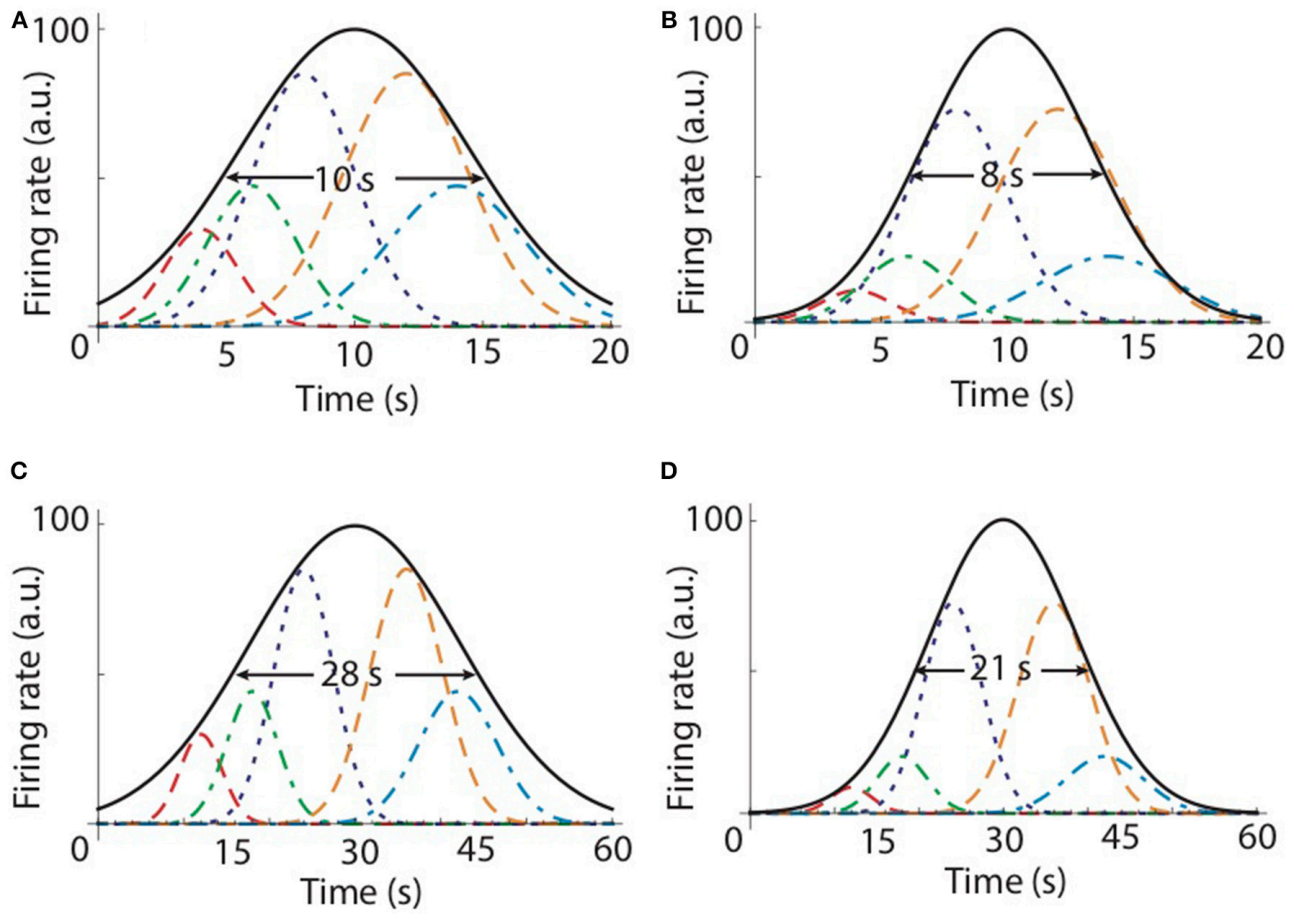
A population of time cells could learn the temporal map for the hippocampus. **(A)** Firing rate curves for five representative time cells that peak at 4 s (red dashed line), 6 s (green dashed-dotted line), 8 s (blue dotted line), 12 s (orange dashed line), and 14 s (slate blue dashed-dotted line), respectively. Starting from an initially uniform distribution of weights, the first learning trial adjusts the weights to 0.15, 0.22, 0.4, 0.4, and 0.22, respectively. **(B)** Based on Equation (3), the second trial further adjusted the weights to 0.02, 0.05, 0.16, 0.16, and 0.05, respectively. The envelope of the population of time cells (black continuous line) shows that the peak is around the to-be-learned criterion time *T* = 10 s and the envelope becomes narrower as the number of trials increases. **(C)** For the criterion time of *T* = 30 s, only some representative firing rate curves are shown for 12 s (red dashed line),18 s (green dashed-dotted line), 24 s (blue dotted line), 36 s (orange dashed line), and 42 s (slate blue dashed-dotted line), respectively. The envelope of the population (black continuous line) peaks at *T* = 30 s. **(D)** The envelope for the second trial is narrower and its width for a criterion time *T* = 30 s **(C,D)** is approximately three times larger compared to width for *T* = 10 s **(A,B)**.

We assume here that the neurobiologically realistic structure sketched in Figure 1B could perform the following computations. Initially, the hippocampus long-term memory stores an arbitrary distribution of weights associated to time cells that peak in the range of the to-be-memorized temporal duration. At the reinforcement time, the striatum coincidence detector updates the weights according to Equation (3), i.e., the time cells that peak closer to the reinforcement time have larger weights compared to those that peak farther. This modulation of time cell activity produces a population response that peaks around the reinforcement time. The weight computation is also flexible enough to ensures the shift of the entire population response to a new reinforcement time. For example, given that the expected outcome should peak at the criterion time *T* = 10 s whereas the actual response in a given trial was at *t* = 8 s, an error signal is generated (see Figure 2). The heuristic mechanism connecting the time of the peak activity of a time cell with the desired behavioral response uses the error signal to modulate the activity of the time cell proportional to its temporal distance to the expected criterion time.

## 3. The model

Based on experimental evidences regarding the existence and the properties of the time cells (Pastalkova et al., 2008; MacDonald et al., 2011; Kraus et al., 2013; Howard et al., 2014), we modeled the activity of each time cell with a Gaussian firing rate curve with the width modulated by its peak time:

(1) $$\begin{array}{r} a_{n}\left(t_{n},\sigma_{n}\right)=A_{n}e^{-\frac{{\left(t-t_{n}\right)}^{2}}{2\sigma_{n}^{2}}}, \end{array}$$

where we assumed for simplicity that the standard deviation σ_*n*_ is proportional to the peak time *t*_*n*_ (see MacDonald et al., 2011 for experimental support of this assumption) and *A*_*n*_ is the maximum firing rate of the time cell with the peak activity at *t*_*n*_.

In agreement with the experimental data (Pastalkova et al., 2008; MacDonald et al., 2011; Kraus et al., 2013; Wang et al., 2015), we assumed that there is a certain, finite, number *N* of time cells that span the entire range of durations required by the behavioral experiment. The error signal (see Figure 1B) modulates the contribution of the individual time cells until the average time field matches as closely as possible the expected outcome. The average time field is determined by the weighted average of the time fields of the *N* time cells:

(2) $$\begin{array}{r} a\left(T_{avg},\sigma_{avg}\right)=\overset{N}{\underset{k\;=\;1}{\sum }}A_{k}e^{-\frac{{\left(t-t_{k}\right)}^{2}}{2\sigma_{k}^{2}}}, \end{array}$$

where *N* is the number of time cells allocated to the current timing task and *A*_*k*_ is the amplitude (weight) associated to an individual time cell. For example, if the expected outcome is *T* = 10 s, then ideally all time cells would have zero weights *A*_*k*_ = 0, except for the cells that peak at *t*_*k*_ = *T* = 10 s. We mathematically modeled the error signal needed in Figure 1C as the difference between the expected outcome *T* and the peak time *t*_*k*_ of each time cell:

(3) $$\begin{array}{r} err_{k}=|T-t_{k}|+\epsilon , \end{array}$$

where ϵ is a very small positive number that helps us avoid a mathematical singularity for the learning rule (Equation 4). At the same time, a small positive ϵ mimics the ubiquitous biological noise. The learning rule is:

(4) $$\begin{array}{r} A_{k}\leftarrow A_{k}/err_{k}, \end{array}$$

where ← indicates that after each learning trial the current weights *A*_*k*_ are replaced by *A*_*k*_/*err*_*k*_. The effect of the above learning rule is that time cells that peak at *t*_*k*_ further from the expected outcome *T* would be stronger depressed compared to those closer to the expected outcome. The weights *A*_*k*_ are stored in a spatially ordered (topological map) memory register that mimics the hippocampal structure (see Figure 1).

## 4. Results

### 4.1. Theoretical predictions regarding the scalar property

There are two distinct contributions to the width of the average time field: (1) the intrinsic properties of individual time field of each time cell, and (2) the global weights learning algorithm.

**Figure 3:**
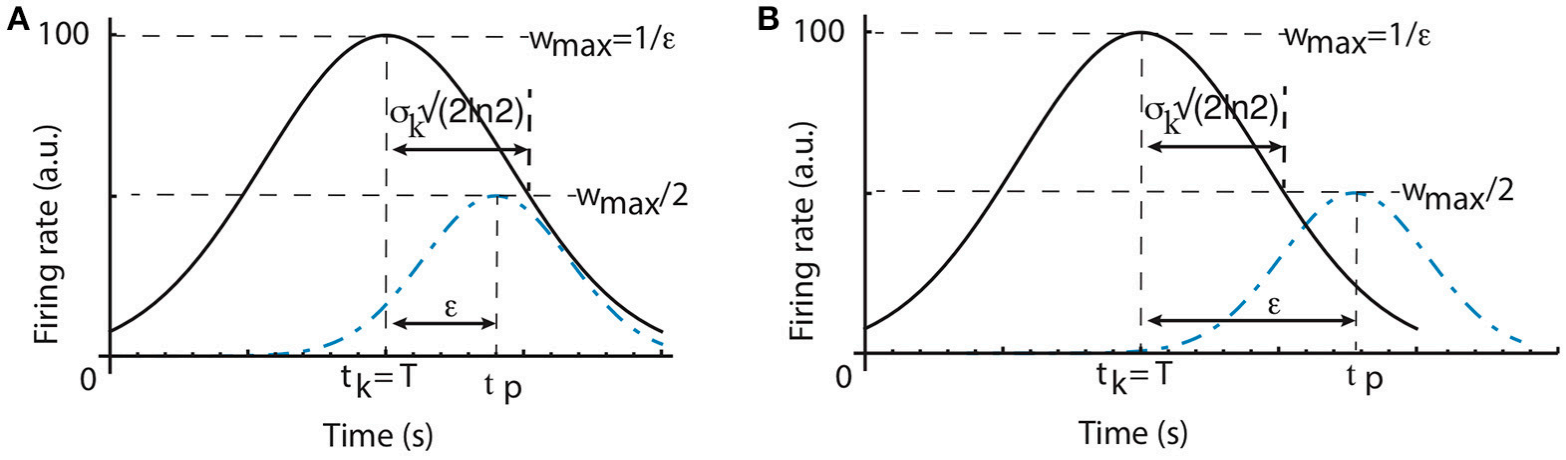
Scalar property predictions. The half-width of the time field of a time cell that peaks at *t*_*k*_, which coincides with the criterion time *T*, is $width_{cell}=\sigma_{k}\sqrt{2\ln2}$ **(A)**. The width of the average time field determined by the learning algorithm is given by the peak time *t*_*p*_ for which $w_{t_{p}}=\frac{1}{2}w_{max}=\frac{1}{2\epsilon }$ (regardless the criterion time). As a result, *width*_*learn*_ = ϵ. If all individual time cell fields are wide enough such that $\sigma_{k}\sqrt{2\ln2}>\epsilon$, then the average time field obeys the scalar property **(A)**. Otherwise, if the time fields of individual time cells are very narrow, then the width of the average time field is only determined by the parameter ϵ of the learning algorithm **(B)**.

**Time Cell Effect on the Width of the Average Time Field**. The half-width of an individual time field of a single cell that peaks at *t*_*k*_ is determined by Equation (1), i.e.,

$$e^{-\frac{{\left(T-t_{k}\right)}^{2}}{2\sigma_{k}^{2}}}=1/2,$$

which gives $width_{cell}=|T-t_{k}|=\sigma_{k}\sqrt{2\ln2}$ as shown in Figure 3.

**Weights Learning Effect on the Width of the Average Time Field**. Even if all time cells were to peak precisely at only one moment in time with no time field width, the average time field still would have a spread determined solely by the learning rule. A time cell that peaks at *t*_*k*_ which exactly overlaps with the criterion time *T* has the maximum possible weight, i.e., $w_{max}=\frac{1}{|T-t_{k}|+\epsilon }=\frac{1}{\epsilon }$ (see Figure 3). To find out the half-width of the average time field due to the weights changing algorithm we need to find the peak activity time *t*_*p*_≠*T* when the weight *w*_*t*_*p*__ is half the maximum weight. Since $w_{t_{p}}=\frac{1}{|T-t_{p}|+\epsilon }$, the width of the average time field due to the learning rule imposed on the weights is:

$$width_{learn}=|T-t_{p}|=\epsilon .$$

As we notice from Figure 3A, it is possible that the intrinsic width of every time field (*width*_*cell*_) is larger than the learning spread *width*_*learn*_. In this case, the width of the average Gaussian time field is determined by the time cell properties. Since the time fields of individual time cells obey the scalar property, it results that the average time field will also obey the same property with exactly the same coefficient of variation as the time cell that is closest to the criterion time. It is also possible to have very narrow time cell fields such that *width*_*cell*_ < *width*_*learn*_ (see Figure 3B). In this case, the width of the average time field is entirely determined by the constant ϵ of the learning rule and the scalar timing is no longer observed. Therefore, depending on the ratio between the intrinsic variance σ_*k*_ of time cell field and the learning rule constant ϵ, it is possible to observe either scalar timing or average time fields of constant width. In practice, we can always select the learning rule constant ϵ such that $\sigma_{k}\sqrt{2\ln2}\;>\;\epsilon$ for all criteria such that the average time field obeys scalar property.

### 4.2. The convergence of the learning algorithm

To emphasize that the width of the individual fields is also proportional to the peak time of the corresponding cells we only showed symmetrically distributed time fields in Figure 2. However, the learning algorithm from Equation (4) converges to the desired criterion time *T* because the learning rule always gives higher weights to cells firing closer (in time) to *T*. Therefore, regardless the initial distribution of weights, they will be repeatedly changed to minimize the error signal from Equation (3), i.e., to reduce the temporal distance of the average time field to the desired criterion time. The weight recalculation only depends on the peak time of the individual time cell *t*_*k*_ and the criterion time *T*. As a result, regardless the distribution of the peak times of time cells (symmetric or not with respect to the criterion time), their weights will always be adjusted to favor cells closer to the criterion time *T*. At the same time, a sparse and asymmetric distributed of time cells could influence the accuracy of timing. For example, for a criterion time of *T* = 10 s it could be that only the following time cells are available: 6, 8, 12, 20, and 30 s, respectively. In this case, although the weights are still correctly calculated to favor the closest time cell to the criterion time of *T* = 10 s, the average time field will peak around 8 s, which leads to a large error compared to the actual criterion time *T* = 10 s. This example suggests that the accuracy of the learning algorithm is determined by the temporal distance of the closest time cell to the criterion time, i.e., *err*_*timing*_ = min{|*T*−*t*_*k*_|}.

### 4.3. Storing multiple criteria with the population-based topological map model

After a set of training trials, the weights stabilize such that the model responds sharply to a criterion time of *T*_1_ = 10 s, i.e., the weights of the time cells near *T*_1_ = 10 s are the largest and they decay with the temporal distance from the desired criterion time. In our implementation of the learning rule given by Equation (4), the weight for a time cell with its peak activity at the desired criterion time *t*_1_ = 10 s is $w_{t_{1}}=\frac{1}{|T_{1}-t_{1}|+\epsilon }=\frac{1}{|T_{1}-10s|+\epsilon }=\frac{1}{\epsilon }$. During the same training trial, a cell that peaks at *t*_2_ = 100 s has a weight of $w_{t_{2}}=\frac{1}{|T_{1}-t_{2}|+\epsilon }=\frac{1}{|T_{1}-100s|+\epsilon }=\frac{1}{90+\epsilon }.$ In other words, the weight of the time cell firing at 100 s is about 90 times weaker $\left(\frac{w_{t_{2}}}{w_{t_{1}}}=\underset{\epsilon \to 0}{\lim}\frac{\epsilon }{90+\epsilon }\approx \frac{1}{90}\right)$ than the one firing at the correct criterion time *T*_1_ = 10 s.

When a new set of training trials starts for a different criterion time *T*_2_ = 100 s, it finds the highest weight $w_{t_{1}}=\frac{1}{\epsilon }$ and changes it to $w_{t_{1}}\leftarrow \frac{w_{t_{1}}}{err_{t_{1}}}=\frac{1}{\epsilon }\frac{1}{|T_{2}-t_{1}|+\epsilon }=\frac{1}{\epsilon \left(90+\epsilon \right)}$. Similarly, at the start of a trial for a new criterion time *T*_2_ the initial weight of $w_{t_{2}}=\frac{1}{90+\epsilon }$ will be changed to $w_{t_{2}}\leftarrow \frac{w_{t_{2}}}{err_{t_{2}}}=\frac{1}{90+\epsilon }\frac{1}{|T_{2}-t_{2}|+\epsilon }=\frac{1}{\epsilon \left(90+\epsilon \right)}$. As we notice, the two weights for the two memorized criteria *T*_1_ = 10 s and *T*_2_ = 100 s remain the largest weights among all memorized values in the hippocampus without requiring any additional counter mechanism to distinguished between *T*_1_ and *T*_2_.

### 4.4. Numerical results

We numerically tested our population-based topological map model and the above learning rule given by Equation (4). We started all numerical simulations with a population of time cells that uniformly covered a time interval that included the criterion time. The range of the peak times covered by the time cells was three times the criterion time. Initially, all time cells have the same maximum firing rate (weight), although we tested that the model works with an arbitrary initial distribution of weights. We tested the model with a variable number of time cells (*N* = 10, 100, and 1,000) and found no difference among the respective average time fields. The criteria tested were *T* = 10 s (see Figures 2A,B) and *T* = 30 s (see Figures 2C,D). In the example shown in Figures 2A,B, we only plotted the firing rate curves of five representative time cells that peak at 4 s (red dashed line), 6 s (green dashed-dotted line), 8 s (blue dotted line), 12 s (orange dashed line), and 14 s (slate blue dashed-dotted line), respectively. After the first learning trial, the weights associated with the time cells were set inversely proportional to the their distance from the to-be-learned criterion time, i.e., *T* = 10 s. Based on our learning rule, for the cells shown in Figure 2, the weights after the first trial became 0.15, 0.22, 0.4, 0.4, and 0.22, respectively (Figure 2A). This shows that the two closest time cells to the to-be-learned criterion time at 8 s and 12 s have the highest weights and all the other weights decrease as the corresponding time cell is farther from the criterion time *T* = 10 s. The second trial further adjusted the weights to 0.02, 0.05, 0.16, 0.16, and 0.05, respectively (Figure 2B). The envelope of the population of time cells (black continuous line in Figure 2) shows that the peak is around the to-be-learned criterion time *T* and the envelope becomes narrower as the number of trials increases. For example, for *T* = 10 s (Figures 2A,B), the half-width of the envelope is 10 s after the first trial and 8 s after the second. Since the weights associated with the contribution of different time cells to the average time field decrease after each trail as a power law, we predict that the width of the average time field will eventually equal the width of the closest time cell to the actual criterion time. We also tested different criteria, such as *T* = 30 s (Figures 2C,D), and found that the half-width of the envelope is indeed approximately three times its value for *T* = 10 s. Therefore, this topological map with time cells and the learning rule given by Equation (4) generates average time fields that follow the scalar property of interval timing.

## 5. Discussion

One of the first and most prominent timing paradigms is the counter model in which a counter keeps track of how many “clock ticks” accumulated since the presentation of a certain salient stimulus that reset the counter (Gibbon, 1977). Later on the counter model was implemented as a drift-diffusion processes with the “clock” represented by some regularly firing neurons (Simen et al., 2011b; Luzardo et al., 2013). A unified model of conditioning and timing that is at its core a drift-diffusion model has been recently developed (Luzardo et al., 2017).

**Difficulties of Counter-Based Models**. One difficulty of the counter models (Gibbon, 1977; Rakitin et al., 1998; Gallistel and Gibbon, 2000) is that they cannot time multiple intervals. For example, in conditioning experiments that rewards the subject either for the first response after a short delay, say 10 s, or the first response after a long delay, say 100 s, the responses were bimodal when the reward is omitted (Catania and Reynolds, 1968; Leak and Gibbon, 1995). However, based on the counter theory, unless the counter model uses different counters for different durations, the output is unimodal with a peak around the average of the two durations. On the other hand, using different counter requires additional information, which is not experimentally available, to identify which counter must be updated (Gallistel, 2007; Machado and Silva, 2007).

**Difficulties of Ramping Firing Models**. Although we used firing rate models to mimic the activity of time cells and to match experimental observations, our population-based time field model does not have the limitations of other firing rate-based models. For example, ramping firing models (Simen et al., 2011b), are limited by the time scale of the leaky integrators. Recently, theoretical approaches suggested that persistently firing neurons (Egorov et al., 2002) could be used to construct time constants up to a few thousand seconds (Tiganj et al., 2015). While a significant improvement, the challenge for the ramping firing model still remains when it comes to covering interval timing processes on a much longer time scale (Howard et al., 2014). A recent solution proposed in Simen et al. (2011a) suggested using a chain of leaky integrators with a decreasing sequence of intrinsic time constants to implement a feedback-based integrator.

**Similarities With SET and LeT Models**. Similar to the SET model, the long-term memory of our previous topological model of the hippocampus (Oprisan et al., 2018) stored a set of reinforcement times, which presumably represent a Gaussian distribution with its peaks around the to-be-learned criterion time. However, we departed from the SET model by assuming in our topological model that the durations are stored in a spatially ordered memory register with short durations stored ventrally and longer durations more dorsal (Oprisan et al., 2018). Such a topological organization was inspired by experimental observations on peak interval shift due to hippocampus lesions. While conceptually straightforward, one drawback of the topological map model is the nature of what is actually stored in the hippocampus. Computational models can store anything, including a floating point number that is the actual reinforcement time of an individual trial (like in SET model). In this paper we suggested a more realistic model of the topological map of the hippocampus by assuming that the memory register actually stores weights associated to individual time cells. This new population-based topological map model of the hippocampus is an improvement over the previous model (Oprisan et al., 2018) in many respects: (1) the properties of the average time field, such as the peak time and width, are directly related to the time field of the time cells, (2) the learning mechanism of the weights ensures that the average time field peaks around the to-be-learned criterion time, and (3) long-term potentiation/depression mechanisms could be invoked for adjusting the weights. The replacement of the memory register content with the weights of the time cells instead of actual reinforcement time is a departure from the SET model and a similarity point with the LeT model. However, in contrast to LeT, in our model the weights are associated to physical time cells of the hippocampus and serve for computing the average time field. At the same time, this new model of a population-based topological map of the hippocampus with time cells is different from LeT weight adjustment model: (1) we did not consider any intrinsic decay rates of the weights as in LeT, and (2) we did not directly relate the weight strengthening to the reinforcement time of individual trials.

**Similarities With Microstimuli Model**. Recently, the microstimuli model (Ludvig et al., 2008) suggested that when a stimulus is presented, it leaves a slowly decaying memory trace, which is encoded by a series of temporal receptive fields. The theory of microstimuli also postulates that the traces with long latency peaks have greater dispersion. Experimental evidences for microstimuli theory have been found in basal ganglia (Adler et al., 2012). They found Gaussian-like post-stimulus time courses (similar to those describing the temporal field of time cells in our model given by Equation 1) in medium spiny neurons recording from the putamen. Similar evidences of the existence of Gauss-like responses were found in caudate nucleus (Jin et al., 2009). While Gauss-like structures of temporal fields have been used by many models from the early SET (Church, 1984; Gibbon and Church, 1984; Gibbon et al., 1984, 1988; Brunner et al., 1997) to the more recent microstimuli (Ludvig et al., 2008), or the time cell temporal field (Pastalkova et al., 2008; MacDonald et al., 2011; Kraus et al., 2013; Wang et al., 2015), our topological map model only apply to the hippocampus. Interval timing has always been thought to involve multiple, complementary, mechanisms served by distributed neural networks. One such distinct mechanism could be the basal ganglia-based microstimuli model.

Here we suggested a novel, population-based, model that uses experimentally verified results regarding the time cells of the hippocampus. The activity of a time cell (1) peaks at a specific time during a timing task, and (2) the firing field width increases with the peak time (Pastalkova et al., 2008; MacDonald et al., 2011; Kraus et al., 2013; Howard et al., 2014). We suggested a possible time perception mechanism which assumes that (1) the hippocampus stores a spatially organized (topological) map of durations (Oprisan et al., 2018) with the added novelty that (2) a memory location in the hippocampus actually stores a pointer to a whole object, which is a time cell defined by the timing of its peak activity and the width of its timing field. Previously, the topological map model introduced in Oprisan et al. (2018) only stored reinforcement time values in a spatially ordered register with smaller durations mapped toward the ventral side and larger durations stored toward the dorsal area of the hippocampus. Such a topological organization accounts for the experimentally observed effect of hippocampus lesions (Yin and Meck, 2014; Tam et al., 2015). While the existence of such topological maps has been supported by experiments, we suggested here a more flexible model that instead of storing actual temporal durations it stored a pointer to a whole object, i.e. a time cell. At the computer implementation level, the topological map actually stores the maximum amplitude (the weight) of the Gaussian firing rate associated to a given time cell. The learning rule for the weights given by Equation (4) accounts for the contribution of individual time cells to the average time field and implements a distance-dependent connectivity, which has experimental support (Perin et al., 2011). Based on this newly proposed learning rule, we found that the average time field based on the topological map of a population of time cells preserves both the Gaussian-like structure and the scalar property of the individual time cell fields.

One obvious implication of this topological map mode with time cells is that the properties of the average time field, i.e., the accuracy of timing and the scalar property, are directly determined by the similar properties of the time cells. As the experiments showed, the firing field of individual time cells is Gaussian and its width scales with the peak time. This cellular-level scalar property is inherited by the average time field, which is a weighted average of individual time fields. Additionally, the shape of the average temporal field is also influenced by the learning rule. Here we adopted a weight adjustment rule based on the temporal distance from the criterion time to the peak time of each individual cell. While experimental support exists for such a distance-based weight computation, other models are possible. Therefore, new experimental data that manipulate the activity of time cells are necessary in order to uniquely identify the learning rule.

This model is only a small piece of a wider picture which attempts to explain how time perception forms in the brain. As it has been suggested, it is likely that there are multiple mechanisms for time perception implemented by a set of distributed neural networks working together toward this goal.

## Author contributions

SO wrote the manuscript and derived the mathematical or computational results. MB and CB reviewed the manuscript.

### Conflict of interest statement

The authors declare that the research was conducted in the absence of any commercial or financial relationships that could be construed as a potential conflict of interest.

## References

[B1] Adler A. Katabi S. Finkes I. Israel Z. Prut Y. Bergman H. (2012). Temporal convergence of dynamic cell assemblies in the striato-pallidal network. J. Neurosci. 32, 2473–2484. 10.1523/JNEUROSCI.4830-11.2012 22396421 PMC6621802

[B2] Atallah H. E. Frank M. J. O'Reilly R. C. (2004). Hippocampus, cortex, and basal ganglia: insights from computational models of complementary learning systems. Neurobiol. Learn. Mem. 82, 253–267. 10.1016/j.nlm.2004.06.004 15464408

[B3] Balsam P. D. Gallistel C. R. (2009). Temporal maps and informativeness in associative learning. Trends Neurosci. 32, 73–78. 10.1016/j.tins.2008.10.004 19136158 PMC2727677

[B4] Bizo L. A. White K. G. (1994). The behavioral theory of timing: reinforcer rate determines pacemaker rate. J. Exp. Anal. Behav. 61, 19–33. 10.1901/jeab.1994.61-19 16812723 PMC1334351

[B5] Brunner D. Fairhurst S. Stolovitsky G. Gibbon J. (1997). Mnemonics for variability: remembering food delay. J. Exp. Psychol. Anim. Behav. Process. 23, 68–83. 9008863 10.1037//0097-7403.23.1.68

[B6] Buhusi C. V. Aziz D. Winslow D. Carter R. E. Swearingen J. E. Buhusi M. C. (2009). Interval timing accuracy and scalar timing in C57BL/6 mice. Behav Neurosci. 123, 1102–1113. 10.1037/a0017106 19824777 PMC2822645

[B7] Buhusi C. V. Meck W. H. (2005). What makes us tick? Functional and neural mechanisms of interval timing. Nat. Rev. Neurosci. 6, 755–765. 10.1038/nrn1764 16163383

[B8] Buhusi C. V. Meck W. H. (2006). Time sharing in rats: a peak-interval procedure with gaps and distracters. Behav. Process. 71, 107–115. 10.1016/j.beproc.2005.11.017 16413701

[B9] Buhusi C. V. Meck W. H. (2009). Relativity theory and time perception: single or multiple clocks? PLoS ONE 4:6268 10.1371/journal.pone.0006268 PMC2707607 19623247

[B10] Buhusi C. V. Meck W. H. (2010). Timing behavior in Encyclopedia of Psychopharmacology, ed Stolerman I. (Berlin: Springer), 1319–1323.

[B11] Buhusi C. V. Oprisan S. A. (2013). Time-scale invariance as an emergent property in a perceptron with realistic, noisy neurons. Behav. Process. 95, 60–70. 10.1016/j.beproc.2013.02.015 23518297 PMC3640655

[B12] Buhusi C. V. Oprisan S. A. Buhusi M. (2016). Clocks within clocks: timing by coincidence detection. Curr. Opin. Behav. Sci. 8, 207–213. 10.1016/j.cobeha.2016.02.024 27004236 PMC4797640

[B13] Buhusi C. V. Paskalis J.-P. G. Cerutti D. (2006). Time-sharing in pigeons: independent effects of gap duration, position and discriminability from the timed signal. Behav. Process. 71, 116–125. 10.1016/j.beproc.2005.10.006 16414210

[B14] Buhusi M. Olsen K. Buhusi C. V. (2017). Increased temporal discounting after chronic stress in chl1-deficient mice is reversed by 5-ht2c agonist ro 60-0175. Neuroscience 357, 110–118. 10.1016/j.neuroscience.2017.05.047 28583411 PMC5546908

[B15] Canal C. Stutz S. Gold P. (2005). Glucose injections into the dorsal hippocampus or dorsolateral striatum of rats prior to t-maze training: modulation of learning rates and strategy selection. Learn. Mem. 12, 367–374. 10.1101/lm.88205 16027177 PMC1183254

[B16] Catania A. C. Reynolds G. S. (1968). A quantitative analysis of the responding maintained by interval schedules of reinforcement. J. Exp. Anal. Behav. 11, 327–383. 10.1901/jeab.1968.11-s327 5672248 PMC1338497

[B17] Christakou A. Robbins T. W. Everitt B. J. (2004). Prefrontal cortical–ventral striatal interactions involved in affective modulation of attentional performance: Implications for corticostriatal circuit function. J. Neurosci. 24, 773–780. 10.1523/JNEUROSCI.0949-03.2004 14749421 PMC6729820

[B18] Church R. M. (1984). Properties of the Internal Clock. New York, NY: New York Academy of Sciences. 10.1111/j.1749-6632.1984.tb23459.x 6588815

[B19] Church R. M. (2003). A Concise Introduction to Scalar Timing Theory. Boca Raton, FL: CRC Press 3–22. 10.1201/9780203009574.sec1

[B20] Church R. M. Broadbent H. A. (1990). Alternative representations of time, number, and rate. Cognition 37, 55–81. 2269008 10.1016/0010-0277(90)90018-f

[B21] Church R. M. Broadbent H. A. (1991). A Connectionist Model of Timing. Hillsdale, NJ: Lawrence Erlbaum Associates.

[B22] Church R. M. Lacourse D. Crystal J. (1998). Temporal search as a function of the variability of interfood intervals. J. Exp. Psychol. Anim. Behav. Process. 24, 291–315. 9679306 10.1037//0097-7403.24.3.291

[B23] Church R. M. Meck W. H. Gibbon J. (1994). Application of scalar timing theory to individual trials. J. Exp. Psychol. Anim. Behav. Process. 20, 135–155. 8189184 10.1037//0097-7403.20.2.135

[B24] Daniels C. W. Sanabria F. (2017). Interval timing under a behavioral microscope: dissociating motivational and timing processes in fixed-interval performance. Learn. Behav. 45, 29–48. 10.3758/s13420-016-0234-1 27443193

[B25] Datta S. Siwek D. F. Patterson E. H. Cipolloni P. B. (1998). Localization of pontine pgo wave generation sites and their anatomical projections in the rat. Synapse 30, 409–423. 10.1002/(SICI)1098-2396(199812)30:4<409::AID-SYN8>3.0.CO;2-# 9826233

[B26] Egorov A. V. Hamam B. N. Fransen E. Hasselmo M. E. Alonso A. A. (2002). Graded persistent activity in entorhinal cortex neurons. Nature 420, 173–178. 10.1038/nature01171 12432392

[B27] Gallistel C. (2007). Flawed foundations of associationism? Comments on machado and silva. Am. Psychol. 62, 682–685. 10.1037/0003-066X.62.7.682 17924751

[B28] Gallistel C. Gibbon J. (2000). Time, rate, and conditioning. Psychol. Rev. 107, 289–344. 10.1037/0033-295X.107.2.289 10789198

[B29] Gallistel C. R. (1990). The Organization of Behavior. Cambridge, MA: MIT Press.

[B30] Garvert M. M. Dolan R. J. Behrens T. E. (2017). A map of abstract relational knowledge in the human hippocampal? Entorhinal cortex. eLife 6:e17086. 10.7554/eLife.17086 28448253 PMC5407855

[B31] Gibbon J. (1977). Scalar expectancy theory and weber's law in animal timing. Psychol. Rev. 84, 279–325. 10.1037/0033-295X.84.3.279

[B32] Gibbon J. (1991). Origins of scalar timing. Learn. Motiv. 22, 3–38. 10.1016/0023-9690(91)90015-Z

[B33] Gibbon J. Church R. M. (1984). Sources of Variance in an Information Processing Theory of Timing. Hillsdale, NJ: Erlbaum.

[B34] Gibbon J. Church R. M. Fairhurst S. Kaceinik A. (1988). Scalar expectancy theory and choice between delayed rewards. Psychol. Rev. 95, 102–114. 3353474 10.1037/0033-295x.95.1.102

[B35] Gibbon J. Church R. M. Meck W. H. (1984). Scalar timing in memory. Ann. N.Y. Acad. Sci. 423, 52–77. 6588812 10.1111/j.1749-6632.1984.tb23417.x

[B36] Gibbon J. Malapani C. Dale C. L. Gallistel C. R. (1997). Toward a neurobiology of temporal cognition: advances and challenges. Curr. Opin. Neurobiol. 7, 170–184. 9142762 10.1016/s0959-4388(97)80005-0

[B37] Glenberg A. Bradley M. M. Stevenson J. A. Kraus T. A. Tkachuk M. J. Gretz A. L. (1980). A two-process account of long-term serial position effects. J. Exp. Psychol. Hum. Learn. Mem. 6, 355–369. 10.1037/0278-7393.6.4.355

[B38] Groenewegen H. J. de Graaf Y. G. Smeets W. J. (1999). Integration and segregation of limbic cortico-striatal loops at the thalamic level: an experimental tracing study in rats. J. Chem. Neuroanat. 16, 167–185. 10.1016/S0891-0618(99)00009-5 10422737

[B39] Gu B. Cheng R. Yin B. Meck W. H. (2011). Quinpirole-induced sensitization to noisy/sparse periodic input: temporal synchronization as a component of obsessive-compulsive disorder. Neuroscience 179, 143–150. 10.1016/j.neuroscience.2011.01.048 21284954

[B40] Harrington D. L. Haaland K. Y. Hermanowicz N. (1998). Temporal processing in the basal ganglia. Neuropsychology 12, 3–12. 9460730 10.1037//0894-4105.12.1.3

[B41] Houk J. (1995). Information processing in modular circuits linking basal ganglia and cerebral cortex in Models of Information Processing in the Basal Ganglia, eds Houk J. Davis J. Beiser D. (Cambridge: MIT Press), 3–10.

[B42] Houk J. Adams J. Barto A. (1995). A Model of How the Basal Ganglia Generate and Use Neural Signals That Predict Reinforcement. Cambridge, MA: MIT Press 249–270

[B43] Howard M. W. MacDonald C. J. Tiganj Z. Shankar K. H. Du Q. Hasselmo M. E.. (2014). A unified mathematical framework for coding time, space, and sequences in the hippocampal region. J. Neurosci. 34, 4692–4707. 10.1523/JNEUROSCI.5808-12.2014 24672015 PMC3965792

[B44] Howard M. W. Youker T. E. Venkatadass V. (2008). The persistence of memory: contiguity effects across several minutes. Psychon. Bull. Rev. 15, 58–63. 10.3758/PBR.15.1.58 18605480 PMC2493616

[B45] Jin D. Z. Fujii N. Graybiel A. M. (2009). Neural representation of time in cortico-basal ganglia circuits. Proc. Natl. Acad. Sci. U.S.A. 106, 19156–19161. 10.1073/pnas.0909881106 19850874 PMC2776432

[B46] Jones C. Jahanshahi M. (2011). Dopamine modulates striato-frontal functioning during temporal processing. Front. Integr. Neurosci. 5:70. 10.3389/fnint.2011.00070 22046150 PMC3200491

[B47] Killeen P. R. Fetterman J. G. (1988). A behavioral theory of timing. Psychol. Rev. 95, 274–295. 3375401 10.1037/0033-295x.95.2.274

[B48] Killeen P. R. Fetterman J. G. (1993). The behavioral theory of timing: transition analyses. J. Exp. Anal. Behav. 59, 411–422. 10.1901/jeab.1993.59-411 8454961 PMC1322052

[B49] Kraus B. J. Robinson R. J. White J. A. Eichenbaum H. Hasselmo M. E. (2013). Hippocampal “time cells”: time versus path integration. Neuron 78, 1090–1101. 10.1016/j.neuron.2013.04.015 23707613 PMC3913731

[B50] Leak T. M. Gibbon J. (1995). Simultaneous timing of multiple intervals: implications of the scalar property. J. Exp. Psychol. Anim. Behav. Process. 21, 3–19. 10.1037/0097-7403.21.1.3 7844504

[B51] Lewis P. A. Miall R. C. (2009). The precision of temporal judgement: milliseconds, many minutes, and beyond. Phil. Trans. R. Soc. Lond. B Biol. Sci. 364, 1897–1905. 10.1098/rstb.2009.0020 19487192 PMC2685820

[B52] Ludvig E. A. Sutton R. S. Kehoe E. J. (2008). Stimulus representation and the timing of reward-prediction errors in models of the dopamine system. Neural Comput. 20, 3034–3054. 10.1162/neco.2008.11-07-654 18624657

[B53] Luzardo A. Alonso E. Mondragon E. (2017). A Rescorla-Wagner drift-diffusion model of conditioning and timing. PLoS Comput. Biol. 13:e1005796. 10.1371/journal.pcbi.1005796 29095819 PMC5685643

[B54] Luzardo A. Ludvig E. A. Rivest F. (2013). An adaptive drift-diffusion model of interval timing dynamics. Behav. Process. 95, 90–99. 10.1016/j.beproc.2013.02.003 23428705 PMC3771865

[B55] MacDonald C. J. Lepage K. Q. Eden U. T. Eichenbaum H. (2011). Hippocampal time cells bridge the gap in memory for discontiguous events. Neuron 71, 737–749. 10.1016/j.neuron.2011.07.012 21867888 PMC3163062

[B56] Machado A. (1997). Learning the temporal dynamics of behavior. Psychol. Rev. 104, 241–265. 9127582 10.1037/0033-295x.104.2.241

[B57] Machado A. Malheiro M. T. Erlhagen W. (2007). Learning to time: a perspective. J. Exp. Anal. Behav. 92, 423–458. 10.1901/jeab.2009.92-423 20514171 PMC2771665

[B58] Machado A. Silva F. J. (2007). Toward a richer view of the scientific method: the role of conceptual analysis. Am. Psychol. 62, 671–681. 10.1037/0003-066X.62.7.671 17924750

[B59] Malapani C. Fairhurst S. (2002). Scalar timing in animals and humans. Learn. Motiv. 33, 156–176. 10.1006/lmot.2001.1105

[B60] Mathis A. Herz A. V. Stemmler M. (2012). Optimal population codes for space: grid cells outperform place cells. Neural Comput. 24, 2280–2317. 10.1162/NECO_a_00319 22594833

[B61] McKenna J. T. Vertes R. P. (2004). Afferent projections to nucleus reuniens of the thalamus. J. Compar. Neurol. 480, 115–142. 10.1002/cne.20342 15514932

[B62] O'Keefe J. (1976). Place units in the hippocampus of the freely moving rat. Exp. Neurol. 51, 78–109. 10.1016/0014-4886(76)90055-8 1261644

[B63] O'Keefe J. Burgess N. (1996). Geometric determinants of the place fields of hippocampal neurons. Nature 381, 425–428. 10.1038/381425a0 8632799

[B64] O'Keefe J. Recce M. L. (1993). Phase relationship between hippocampal place units and the EEG theta rhythm. Hippocampus 3, 317–330. 10.1002/hipo.450030307 8353611

[B65] Oprisan S. A. Aft T. Buhusi M. Buhusi C. V. (2018). Scalar timing in memory: a temporal map in the hippocampus. J. Theor. Biol. 438, 133–142. 10.1016/j.jtbi.2017.11.012 29155279 PMC6432786

[B66] Oprisan S. A. Buhusi C. V. (2011). Modeling pharmacological clock and memory patterns of interval timing in a striatal beat-frequency model with realistic, noisy neurons. Front. Integr. Neurosci. 5:52. 10.3389/fnint.2011.00052 21977014 PMC3178804

[B67] Oprisan S. A. Buhusi C. V. (2013). How noise contributes to time-scale invariance of interval timing. Phys. Rev. E 87:052717. 10.1103/PhysRevE.87.052717 23767576 PMC7015149

[B68] Oprisan S. A. Buhusi C. V. (2014). What is all the noise about in interval timing? Philos. Trans. R. Soc. Lond. B Biol. Sci. 369:20120459. 10.1098/rstb.2012.0459 24446493 PMC3895984

[B69] Packard M. Cahill L. McGaugh J. (1994). Amygdala modulation of hippocampal-dependent and caudate nucleus-dependent memory processes. Proc. Natl. Acad. Sci. U.S.A. 91, 8477–8481. 8078906 10.1073/pnas.91.18.8477 PMC44629

[B70] Parent A. Hazrati L.-N. (1995a). Functional anatomy of the basal ganglia. I. The cortico-basal ganglia-thalamo-cortical loop. Brain Res. Rev. 20, 91–127. 10.1016/0165-0173(94)00007-C 7711769

[B71] Parent A. Hazrati L.-N. (1995b). Functional anatomy of the basal ganglia. II. The place of subthalamic nucleus and external pallidium in basal ganglia circuitry. Brain Res. Rev. 20, 128–154. 10.1016/0165-0173(94)00008-D 7711765

[B72] Pastalkova E. Itskov V. Amarasingham A. Buzsaki G. (2008). Internally generated cell assembly sequences in the rat hippocampus. Science 321, 1322–1327. 10.1126/science.1159775 18772431 PMC2570043

[B73] Penner M. Mizumori S. (2012). Age-associated changes in the hippocampal-ventral striatum-ventral tegmental loop that impact learning, prediction, and context discrimination. Front. Aging Neurosci. 4:22. 10.3389/fnagi.2012.00022 22891060 PMC3413901

[B74] Perin R. Berger T. K. Markram H. (2011). A synaptic organizing principle for cortical neuronal groups. Proc. Natl. Acad. Sci. U.S.A. 108, 5419–5424. 10.1073/pnas.1016051108 21383177 PMC3069183

[B75] Rakitin B. C. Gibbon J. Penney T. B. Malapani C. Hinton S. C. Meck W. H. (1998). Scalar expectancy theory and peak-interval timing in humans. J. Exp. Psychol. Anim. Behav. Process. 24, 15–33. 10.1037/0097-7403.24.1.15 9438963

[B76] Schapiro A. C. Turk-Browne N. B. Norman K. A. Botvinick M. M. (2015). Statistical learning of temporal community structure in the hippocampus. Hippocampus 26, 3–8. 10.1002/hipo.22523 26332666 PMC4715493

[B77] Schiller D. Eichenbaum H. Buffalo E. A. Davachi L. Foster D. J. Leutgeb S.. (2015). Memory and space: towards an understanding of the cognitive map. J. Neurosci. 35, 13904–13911. 10.1523/JNEUROSCI.2618-15.2015 26468191 PMC6608181

[B78] Schultz W. (2002). Getting formal with dopamine and reward. Neuron 36, 241–263. 10.1016/S0896-6273(02)00967-4 12383780

[B79] Silkis I. (2008). A mechanism for influencing the septo-hippocampal theta rhythm by dopamine through the basal ganglia. Neurochem. J. 2, 157–163. 10.1134/S1819712408030045

[B80] Simen P. Balci F. deSouza L. Cohen J. Holmes P. (2011a). Interval timing by long-range temporal integration. Front. Integr. Neurosci. 5:28. 10.3389/fnint.2011.00028 21747762 PMC3130150

[B81] Simen P. Balci F. deSouza L. Cohen J. D. Holmes P. (2011b). A model of interval timing by neural integration. J. Neurosci. 31, 9238–9253. 10.1523/JNEUROSCI.3121-10.2011 21697374 PMC3142662

[B82] Tam S. Bonardi C. (2012a). Dorsal hippocampal involvement in appetitive trace conditioning and interval timing. Behav. Neurosci. 126, 258–269. 10.1037/a0027164 22352787

[B83] Tam S. Bonardi C. (2012b). Dorsal hippocampal lesions disrupt pavlovian delay conditioning and conditioned-response timing. Behav. Brain Res. 230, 259–267. 10.1016/j.bbr.2012.02.016 22366272

[B84] Tam S. Jennings D. Bonardi C. (2013). Dorsal hippocampal involvement in conditioned-response timing and maintenance of temporal information in the absence of the CS. Exp. Brain Res. 227, 547–559. 10.1007/s00221-013-3530-4 23652722

[B85] Tam S. Jennings D. Bonardi C. (2015). Effects of dorsal hippocampal damage on conditioning and conditioned-response timing: A pooled analysis. Hippocampus 25, 444–459. 10.1002/hipo.22381 25331034

[B86] Teki S. Grube M. Griffiths T. (2012). A unified model of time perception accounts for duration-based and beat-based timing mechanisms. Front. Integr. Neurosci. 5:90. 10.3389/fnint.2011.00090 22319477 PMC3249611

[B87] Thierry A.-M. Gioanni Y. Degenetais E. Glowinski J. (2000). Hippocampo-prefrontal cortex pathway: anatomical and electrophysiological characteristics. Hippocampus 10, 411–419. 10.1002/1098-1063(2000)10:4<411::AID-HIPO7>3.0.CO;2-A 10985280

[B88] Tiganj Z. Hasselmo M. Howard M. (2015). A simple biophysically plausible model for long time constants in single neurons. Hippocampus 25, 27–37. 10.1002/hipo.22347 25113022 PMC4437581

[B89] Vertes R. P. (2001). Analysis of projections from the medial prefrontal cortex to the thalamus in the rat, with emphasis on nucleus reuniens. J. Compar. Neurol. 442, 163–187. 10.1002/cne.10083 11754169

[B90] Voorn P. Vanderschuren L. Groenewegen H. Robbins T. Pennartz C. (2004). Putting a spin on the dorsal-ventral divide of the striatum. Trends Neurosci. 27, 468–474. 10.1016/j.tins.2004.06.006 15271494

[B91] Wang Y. Romani S. Lustig B. Leonardo A. Pastalkova E. (2015). Theta sequences are essential for internally generated hippocampal firing fields. Nat. Neurosci. 18, 282–288. 10.1038/nn.3904 25531571

[B92] Yin B. Meck W. H. (2014). Comparison of interval timing behaviour in mice following dorsal or ventral hippocampal lesions with mice having d-opioid receptor gene deletion. Phil. Trans. R. Soc. B 369:20120466. 10.1098/rstb.2012.0466 24446500 PMC3895991

